# Adaptation costs to constant and alternating polluted environments

**DOI:** 10.1111/eva.12510

**Published:** 2017-11-10

**Authors:** Morgan Dutilleul, Denis Réale, Benoit Goussen, Catherine Lecomte, Simon Galas, Jean‐Marc Bonzom

**Affiliations:** ^1^ Laboratoire d’écotoxicologie des radionucléides Institut de Radioprotection et de Sûreté Nucléaire, Cadarache Saint‐Paul‐lez‐Durance Cedex France; ^2^ Département des Sciences Biologiques Université du Québec À Montréal Montréal QC Canada; ^3^ Faculté de pharmacie Laboratoire de Toxicologie Université de Montpellier 1 Montpellier Cedex 5 France; ^4^ Unit “Models for ecotoxicology and toxicology” (METO) INERIS Parc ALATA Verneuil‐en‐Halatte France; ^5^ Present address: Environment Department University of York Heslington York UK; ^6^ Present address: Safety and Environmental Assurance Centre Unilever Sharnbrook Bedfordshire UK

**Keywords:** adaptation costs, *Caenorhabditis elegans*, evolution towards generalism, experimental evolution, life history strategy, pollution, resistance, salt, uranium

## Abstract

Some populations quickly adapt to strong and novel selection pressures caused by anthropogenic stressors. However, this short‐term evolutionary response to novel and harsh environmental conditions may lead to adaptation costs, and evaluating these costs is important if we want to understand the evolution of resistance to anthropogenic stressors. In this experimental evolution study, we exposed *Caenorhabditis elegans* populations to uranium (U populations), salt (NaCl populations) and alternating uranium/salt treatments (U/NaCl populations) and to a control environment (C populations), over 22 generations. In parallel, we ran common‐garden and reciprocal‐transplant experiments to assess the adaptive costs for populations that have evolved in the different environmental conditions. Our results showed rapid evolutionary changes in life history characteristics of populations exposed to the different pollution regimes. Furthermore, adaptive costs depended on the type of pollutant: pollution‐adapted populations had lower fitness than C populations, when the populations were returned to their original environment. Fitness in uranium environments was lower for NaCl populations than for U populations. In contrast, fitness in salt environments was similar between U and NaCl populations. Moreover, fitness of U/NaCl populations showed similar or higher fitness in both the uranium and the salt environments compared to populations adapted to constant uranium or salt environments. Our results show that adaptive evolution to a particular stressor can lead to either adaptive costs or benefits once in contact with another stressor. Furthermore, we did not find any evidence that adaptation to alternating stressors was associated with additional adaption costs. This study highlights the need to incorporate adaptive cost assessments when undertaking ecological risk assessments of pollutants.

## 1 INTRODUCTION

Environmental changes, such as pollution or habitat fragmentation, have increased in frequency and intensity throughout the world as the result of anthropogenic activities (Millennium Ecosystem Assessment, 2005). Understanding evolutionary responses to these changes may be critical for the conservation of natural populations in the future (Bell & Collins, 2008; Tilman & Lehman, 2001). Adaptive genetic variation should allow populations to quickly adapt to severe and novel stressors, and thus reduce their risk of extinction (Bell & Collins, 2008; Charlesworth & Hughes, 2000; Hoffmann & Parsons, 1991; Reed, Lowe, Briscoe, & Frankham, 2003). For instance, some populations have been shown to evolve rapidly in response to several pollutants, such as xenobiotics or heavy metals (Jansen, Stoks, Coors, van Doorslaer, & de Meester, 2011; Lopes, Sucena, Santos, & Magalhães, 2008; Salice, Anderson, & Roesijadi, 2010; Shirley & Sibly, 1999; Ward & Robinson, 2005; Xie & Klerks, 2003), and there is strong evidence that adaptive changes in response to selection in a given environment can happen over just a few generations (Hoffmann & Parsons, 1991; Morgan, Kille, & Stürzenbaum, 2007). However, such adaptive response is often hypothesized to come with a cost that constrains future evolutionary potential in several ways (Bergelson & Purrington, 1996; Coustau, Chevillon, & ffrench‐Constant, 2000). Firstly, rapid adaptation can be associated with a reduction in genetic diversity (Athrey, Leberg, & Klerks, 2007; Ward & Robinson, 2005), which may restrict the population from dealing with future selection pressures (Jansen, Stoks, et al., 2011; Salice et al., 2010; Xie & Klerks, 2003). Secondly, antagonistic pleiotropy creates genetic trade‐offs that can limit the evolutionary potential of a population in rapidly changing environments (Fry, 1993; Williams, 1957). Finally, the evolution of specific life history strategies in response to a novel environment may not confer a selective advantage in either the former or future novel environments (Guedes, Oliveira, Guedes, Ribeiro, & Serrão, 2006; Sibly & Calow, 1989).

Adaptation costs are generally expected when a population that has adapted to a particular stressor has to deal with another novel stressor (Jansen, Stoks, et al., 2011; Mireji et al., 2010; Shirley & Sibly, 1999; Ward & Robinson, 2005; Xie & Klerks, 2003). However, in some studies, adaptation to a particular stressor has been found to be beneficial to the population (Arnaud & Haubruge, 2002; Ward & Robinson, 2005; Xie & Klerks, 2003) or to entail no cost or benefit (Coustau et al., 2000; Lopes et al., 2008; McCart, Buckling, & ffrench‐Constant, 2005; Reznick, Nunney, & Tessier, 2000), once in the presence of other stressors. Results failing to show costs to alternative environments have been attributed to the difficulty in statistically detecting antagonistic pleiotropy or to the choice of the environmental conditions by those studies that did not produce any cost. An example of the different cases is described in the study of Ward and Robinson (2005): after adapting to cadmium, *Daphnia magna* populations have shown costs to phenol, but equivalent fitness when exposed to copper and higher fitness when exposed to lead. Despite their strong significance for evolutionary and conservation biology, it is not clear yet how generalizable adaption costs and benefits are, and in which conditions they occur.

Experimental studies on adaptation costs generally focus on costs induced by a constant stress from a single stressor (Jansen, Stoks, et al., 2011; Ward & Robinson, 2005; Xie & Klerks, 2003) or from a simultaneous combination of stressors (Jansen, de Meester, Cielen, Buser, & Stoks, 2011; Jasmin & Kassen, 2007; Koskella, Lin, Buckling, & Thompson, 2012). Comparatively few studies have examined how populations adapt to a temporally heterogeneous environment and its consequence on adaptation costs (Magalhães, Cailleau, Blanchet, & Olivieri, 2014; Reed et al., 2003; Turner & Elena, 2000), despite the fact that wild populations experience temporal environmental heterogeneity (Hedrick, 1986; Levins, 1968). Populations may not adapt as quickly in a temporally heterogeneous environment, with multiple successive stressors, than in an environment characterized by a single stressor (but see Turner & Elena, 2000). However, environmental heterogeneity may help populations maintain a higher level of genetic variation (Hedrick, 1986; Roff, 2002), and lower adaptation costs in comparison with evolution in a homogeneous environment (Reed et al., 2003). Predictions of the adaptation costs in heterogeneous environments relative to those in homogeneous environments are not clear yet, and it is necessary to assess their respective effects on the evolution of adaptation costs.

In this study, we used an experimental evolution approach with *Caenorhabditis elegans* populations to test whether 1) adaptive evolution to a particular stressor leads to adaptation costs when the population is transferred into a nonpolluted environment or has to deal with another stressor; and 2) adaptive evolution in a heterogeneous environment leads to higher or lower adaptation costs than evolution in homogeneous environments. Populations were allowed to evolve for 22 generations in response to a constant uranium environment (U populations), a constant high sodium chloride environment (NaCl populations) or an alternating U/NaCl environment at each generation (U/NaCl populations). A set of control populations (C populations) was maintained for the same number of generations in the same environment without any stressor. Uranium is a natural radioactive heavy metal whose concentrations in sediments or surface soils have increased recently as a result of human activities, such as mining (Lottermoser, Ashley, & Costelloe, 2005; UNSCEAR, 2000). Exposure to natural uranium may induce both chemical and radiological effects, although uranium is assumed to have higher chemotoxic than radiotoxic effects (Mathews et al., 2009; Miller, Stewart, Brooks, Shi, & Page, 2002). It accumulates in the cells and affects the intestinal epithelium (Giovanetti, Fesenko, Cozzella, Asencio, & Sansone, 2010), thus reducing energy and nutrient assimilation. In *C. elegans*, uranium is assumed to decrease the assimilation of energy from food (Goussen et al., 2015). Salt concentration has recently increased in several ecosystems with important sources of salt originating from winter road maintenance, wastewater and intensive irrigation (e.g., Dugan et al., 2017; Müller & Gächter, 2012; Rengasamy, 2006; Verwey & Vermeulen, 2011). High salt exposure is an extreme hypertonic stress that provokes a rapid water and solute content loss in *C. elegans* cells (Lamitina, Morrison, Moeckel, & Strange, 2004).

We chose to study life history (growth, early and late fertility) and behaviour traits (male body bend) that are directly or indirectly linked to fitness and to the dynamics of the populations (Dutilleul et al., 2014). These traits are also generally phenotypically correlated with each other, are involved into particular life history strategies (Pigliucci & Preston, 2004) and thus may coevolve in response to a given stressor. We ran two types of experiments with the populations from the selection experiment (see Fig. A1 in Appendix S1 for a schematic representation). First, we transferred individuals from U, NaCl or U/NaCl populations back into the original environmental conditions and compared their fitness with that of individuals from control populations (i.e., common‐garden experiment [CG]; Conover & Schultz, 1995; see Fig. A2 in Appendix S1). The CG experiments allowed us to test whether adaptation to a particular pollutant incurs adaptation costs when the population experiences a nonpolluted environment. A lower population fitness compared to the control populations would show the adaptation costs of the populations that evolved in the polluted environments. Successive common‐garden experiments at generations 6, 9, 12, 15 and 18 of the selection experiment allowed us to analyse the evolutionary dynamics of potential adaptation costs (Brausch & Smith, 2009). Second, at generation 18, we transferred individuals from the control, U, NaCl or U/NaCl populations into either the NaCl or the U environment and compared their fitness (i.e., reciprocal‐transplant experiment [RT], Hassel, Pedersen, & Söderström, 2005; Iraeta, Monasterio, Salvador, & Díaz, 2006; see Fig. A3 in Appendix S1). This RT experiment allowed us to test whether adaptation to a particular pollutant incurs adaptation costs when the population is subjected to another stressor. We predicted that adaptation costs would be revealed by a decrease in fitness in the transplant environment compared to the evolved environment. Using CG and RT experiments, we also tested whether adaptation costs differed between heterogeneous (i.e., U/NaCl) and homogenous environments (U or NaCl). We predicted higher adaptation costs for the populations adapted to the alternating U/NaCl environment than for the populations in homogeneous polluted environments (U or NaCl).

## 2 MATERIAL AND METHODS

### 2.1 Population maintenance

*Caenorhabditis elegans* is a good metazoan model and is widely used in microevolution experiments because of its short life cycle, small body length and ease of handling (Braendle, Milloz, & Félix, 2008). *Caenorhabditis elegans* is an androdioecious organism (i.e., self‐fertilization of hermaphrodites and facultative outcross with males). We used a stock population of *C. elegans*, composed of a mixture of 16 wild isolates, so that we could obtain a study population with a large genetic diversity (Teotónio, Carvalho, Manoel, Roque, & Chelo, 2012). This composite population ensured that its genetic architecture did not result from past selection pressures caused by the presence of a pollutant. Therefore, we can be confident that the study population was not previously adapted to the treatment environments it was submitted to. The population was kept in the experimental conditions described in Teotónio et al. (2012) for over 140 generations prior to our study, and about 30% of individuals were male.

At the start of our study, we placed 500 individuals in a 9‐cm‐diameter Petri plate filled with an agar medium seeded with one ml UV‐killed *Escherichia coli* (OP50 strain) as a food source. The generation time for *C. elegans* (i.e., time to complete a life cycle) is <3 days (Byerly, Cassada, & Russell, 1976), so after 3 days of culturing, we transferred 500 individuals at all developmental stages into a new Petri plate for a total of six replicated populations (see Dutilleul et al., 2014 for more details about protocol changes). The nematodes were cultured, throughout the experiment, at 20°C and 80% relative humidity.

### 2.2 Selection experiment

After maintaining the stock populations in our laboratory over 40 generations following the protocol described above, the individuals from the six replicates were mixed and transferred into four different environmental conditions: a control environment and three polluted environments, which were identical to the control in all aspects, except that the agar medium also contained (i) 1.1 mM of uranium (uranyl nitrate: UO_2_ (NO_3_)_2_, 6H_2_O; Sigma‐Aldrich, France), (ii) 308 mM NaCl or (iii) the same concentrations of uranium and salt alternating at each generation (salt for odd generations). We have previously extensively described how we added the pollutant to the agar medium (Dutilleul et al., 2014). For each environmental treatment and the control environment, we created six replicate populations of 500 individuals each, and these populations were transferred into a new Petri dish every 3 days. The uranium and salt concentrations reduced fertility by about 60% after the first generation of exposure.

For the selection experiment, we report the effects of the different selection regimes on fitness at generations 1, 4 and 22 (i.e., the end) of the experiment. Before generation 4, part of the changes in the traits between two successive generations could be attributable to intra‐ and cross‐generational (i.e., parental effects) phenotypic plastic response to the novel environment (Mousseau & Fox, 1998; Räsänen & Kruuk, 2007; Scheiner, 1993). From generation 4 onwards, we assumed that changes caused by nongenetic responses to the novel environment were negligible, and all the observed changes across generations could be explained by genetic changes in response to selection (Dutilleul et al., 2013). Generation time varied between treatments. The NaCl treatment, in particular, delayed generation time compared to the other treatments. Each experimental iteration (i.e., 3 days) may therefore correspond to either a generation or slightly less than a generation. However, for the sake of simplicity, we have used the term “generation” throughout the text.

### 2.3 Common‐garden and reciprocal‐transplant experiments

We estimated adaptation costs to uranium (U), to salt (NaCl) and to the alternating treatment (U/NaCl), respectively. Prior to measuring the traits (see section Measured life history traits), we kept the different populations in their novel environment (i.e., nonpolluted environment in common‐garden and U or NaCl environment in reciprocal‐transplant experiment) for three generations to ensure that the differences between populations only reflected genetic differentiation between the populations and not parental effects (Kawecki et al., 2012; Mousseau, Uller, Wapstra, & Badyaev, 2009).

Starting from generation 6 in the selection experiment, and then every three generations, we ran a CG experiment by isolating 500 individuals from each replicate and putting them in the control environment (Fig. A2 in Appendix S1). Genetic changes caused by adaptation to a specific treatment are reflected by phenotypic differences between the populations when they are exposed to the same environment (Conover & Schultz, 1995; Levins, 1968). For example, a decrease in total fertility for populations that have evolved in the polluted environment compared to the control populations indicated that adaptation costs had been incurred during adaption to that pollutant.

At generation 18, we ran an RT experiment in which samples of each replicate population for each treatment were transferred into both U and NaCl environments (see Fig. A3 in Appendix S1 for a schematic representation). Adaptation costs should be revealed by a negative interaction between the treatment (i.e., the environment in which the population has evolved during the selection experiment) and the transplant environment (see below: statistical analyses). To simplify, populations maintained in the same environment during the selection experiment and the RT experiment (e.g., the NaCl‐adapted populations transferred in the transplant to NaCl) are also considered as “transplanted populations.”

### 2.4 Measured life history traits

At the end of each generation preceding the measurements of traits (see sections Selection experiment/Common‐garden and reciprocal‐transplant experiments), we transferred 100 eggs per replicate to another Petri plate containing the same medium. Time at the transplant was recorded as *t *=* *0. After 48 hr, we counted the number individuals alive and determined their sex. We then randomly picked three hermaphrodites per replicate (i.e., 18 hermaphrodites per treatment) and measured their early (i.e., before 96 hr) and late (i.e., after 96 hr) brood sizes (hereafter referred to as early and late fertility, respectively). We assumed that a reduction of early fertility with an increase in late fertility was an indicator of a longer generation time and conversely that an increase in early fertility and decrease in late fertility revealed a shorter generation time. We calculated total fertility during the overall life of each hermaphrodite as the sum of early and late brood size. We also measured body bend frequency for three males per replicate at 96 hr (body bend frequency is also measurable in hermaphrodite individuals). Body bend frequency reflects speed during locomotion (Tsalik & Hobert, 2003). At age 96 hr, the individuals that had been tested for fertility and body bend frequency were photographed using a stereomicroscope (Olympus SZX12, 1.6 × 90 magnification) with a computer‐connected camera (Nikon D5000). These pictures enabled us to measure body length, which we used as an index of growth from age 0 to 96 hr. All the traits were measured in the selection, the RT and the CG experiments. Detailed results on the selection experiment were reported elsewhere (Dutilleul et al., 2014), and here, we only show results on fitness (i.e., the product of total fertility by the survival rate for each replicate) for this experiment. Survival was affected by the treatment in the selection experiment, but not in the CG and RT experiments. Moreover, survival was not affected by the interaction between the treatment and the generation in the CG experiments or between the treatment and the transplant environment in the RT experiment (see Fig. C and Tables C1, C2 in Appendix S3). We thus used total fertility alone as an index of fitness in the CG and RT experiments. More details about the medium conditions, quantity of food supplied and the measurements are available in our previous study (Dutilleul et al., 2014).

### 2.5 Statistical analysis

We used linear mixed effects models (LMMs) that were implemented within a Bayesian Monte Carlo Markov chain (MCMC) framework (MCMCglmm package; Hadfield, 2010) in the R software (R Development Core Team, 2012). We compared the different treatments using separate statistical models for hermaphrodites and males.

We first tested for the effects of environment and generation (i.e., generations 1, 4 and 22) and their interaction on fitness (i.e., fertility multiplied by survival frequency) measured during the selection experiment. Replicate was used as a random effect to control for potential pseudo‐replication. CG experimental data were used to test for the effects of treatment (i.e., the environment in which the population had evolved), generation (i.e., the generation at which the experiment was performed) and their interaction on hermaphrodite traits (i.e., early, late and total fertility, growth rate) and on male traits (i.e., growth rate and body bend frequency). We used quadrivariate and bivariate models for hermaphrodite and male traits, respectively. RT experimental data were used to test for the effects of the transplant environment as a fixed effect.

We chose a Gaussian distribution for all the traits. For the multivariate analyses, we ran a model that estimated covariance between pairs of traits and a model where these covariance components were fixed to zero. These two models differ in the fact that the traits are assumed to be genetically associated or independent of each other, respectively. To avoid any bias in the results caused by mean trait differences, we rescaled the traits prior to analysis by subtracting each value from the mean of the sample and dividing it by twice the standard deviation (Gelman, 2008). We retained a slightly informative but proper prior (ν = *k* − 1 + 0.002) with a low variance parameter (*V* = diag(*k*)*V_p_*0.05), where *V*_p_ is the phenotypic variance and *k* is the dimension of *V* (e.g., number of traits). After checking for the convergence of parameters values (i.e., number of iterations, burn‐in phase and thinning) and autocorrelation, we retained 120,000 iterations with a burn‐in phase of 20,000, for a total of 1,000 samples for each analysis (Hadfield, 2010).

Among the different fixed effects models, the best‐fitting model had the lowest deviance information criterion (DIC) and the lowest number of parameters when its DIC was within five points of the next best‐fitting model (Spiegelhalter, Thomas, Best, & Lunn, 2007). In addition, if the model had a 95% highest posterior density interval (HPDI) for a fixed effect that did not overlap with zero, we considered it as supplementary evidence that the effect was truly significant. When comparing a trait under two conditions, we also checked whether the 95% HPDI of the difference between the whole posterior distributions of the trait for the two conditions overlapped 0.

## 3 RESULTS

Differences among replicate populations represented between 0% and 5.2% of the overall variance in the traits included in the selected models for the selection, the CG and the RT experiments (Tables 1, 2, 3), which suggested that the sampling of the founder individuals in the different populations was random.

**Table 1:** Comparison (deviance information criterion) between univariate mixed models for hermaphrodite fitness (fertility multiplied by survival frequency) as a function of environment (control, uranium, salt or alternating U/NaCl treatment), generation (1st, 4th and 22nd generation) and their interaction, in a selection experiment

Effect included within the model	DIC	Δ DIC
For hermaphrodite fitness		
–	712.809	–
Environment	677.319	−35.490
Environment + generation	652.104	−25.215
**Environment × generation**	**622.449**	−**29.655**
Replicates effect: 0.0%		

The total variance percentage of fitness explained by replicate random effects is shown at the bottom of the table. The retained model is in bold.

**Table 2:** Comparison (deviance information criterion) between multivariate mixed models for hermaphrodite traits (total, early and late fertility, and growth) or male traits (growth and body bend frequency) measured in common‐garden experiments in a control environment at generations 6, 9, 12, 15 and 18

Effect included within the model	DIC	Δ DIC
For hermaphrodite traits		
–	−704.829	–
Treatment	−721.353	−16.524
Treatment + generation	−739.623	−18.270
**Treatment × generation**	−**744.419**	−**4.796**
Treatment** × **generation (no cov)	1585.925	2330.344
For male traits		
–	1046.823	–
Treatment environment	1041.722	−5.101
**Treatment + generation**	**1023.955**	−**17.767**
Treatment** × **generation	1025.881	1.926
Treatment** × **generation (no cov)	1024.779	−1.102
Replicates effect: 2.3% (hermaphrodites) and 2.0% (males)		

Treatment is the environment in which the population has evolved (i.e., control, uranium, salt or alternating U/NaCl treatment). The total variance percentage of a trait explained by replicate random effects is shown at the bottom of the table. The retained models are in bold.

**Table 3:** Comparison (deviance information criterion) between models for hermaphrodite traits (total, early and late fertility, and growth) or male traits (growth and body bend frequency) measured in the reciprocal‐transplant experiment at generation 18

Effect included within the model	DIC	Δ DIC
For hermaphrodite traits		
–	−654.464	–
Transplant environment	−1101.912	−447.448
Transplant environment + treatment	−1117.402	−15.490
**Transplant environment × treatment**	−**1172.517**	−**55.115**
Transplant environment** × **treatment (no cov)	−2.699	1169.818
For male traits		
–	600.821	–
Transplant environment	422.846	−177.975
Transplant environment + treatment	401.901	−20.945
Transplant environment** × **treatment	378.241	−23.660
**Transplant environment × treatment (no cov)**	**376.365**	−**1.876**
Replicates effect: 5.2% (hermaphrodites) and 3.0% (males)		

Treatment is the environment in which the population has evolved (i.e., control, uranium, salt or alternating U/NaCl treatment), and transplant environment corresponds to the environment to which the populations were transplanted. The total variance percentage of a trait explained by replicate random effects is shown at the bottom of the table. The retained models are in bold.

### 3.1 Selection experiment

**Figure 1:**
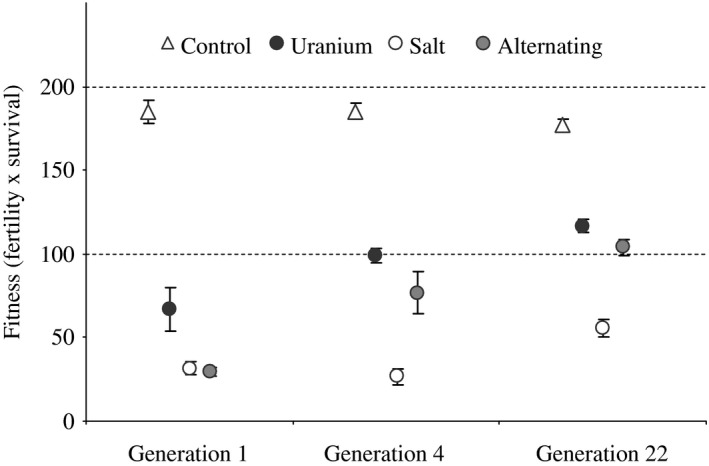
Fitness (i.e., total fertility** × **survival) of hermaphrodite *Caenorhabditis elegans*, in their current environment, at generations 1, 4 and 22 in the selection experiment. Symbols represent means and standard errors of the trait over the six replicated populations in each treatment (control* *=* *empty triangle, uranium* *=* *filled black dots, salt* *=* *empty dots, alternating U/NaCl treatment* *=* *filled grey dots). In the alternating U/NaCl treatment, populations were exposed to NaCl every odd generation (i.e., generation 1 here)

The best‐fitting model for fitness included the interaction between environment and generation, which described the changes in fitness over time and their relationship with particular environments (Table 1). Fitness did not change significantly over time in the control environment (fitness in C at generation 1: 184.9 with 95% HPDI* *=* *[171.1; 197.4], fitness in C at generation 4 minus generation 1: 4.3 [−19.2; 19.9] and 22 minus 4: −10.5 [−26.0; 12.3]; Figure 1). In contrast, in the polluted environments, populations showed a decrease in fitness at generation 1 (fitness between treatments, C minus U: −118.9 with 95% HPDI* *=* *[−135.9; −99.3], C minus NaCl: −156.1 [−172.2; −133.6], C minus U/NaCl: −154.0 [−174.6; −136.7]), followed by a strong increase in fitness over time (Figure 1). Respectively in the uranium, salt and alternating treatments, the gains between generations 4 and 22 were 17.3 [0.2; 36.0], 26.9 [9.6; 47.7], 27.9 [10.3; 46.4].

### 3.2 Common‐garden experiments

**Figure 2:**
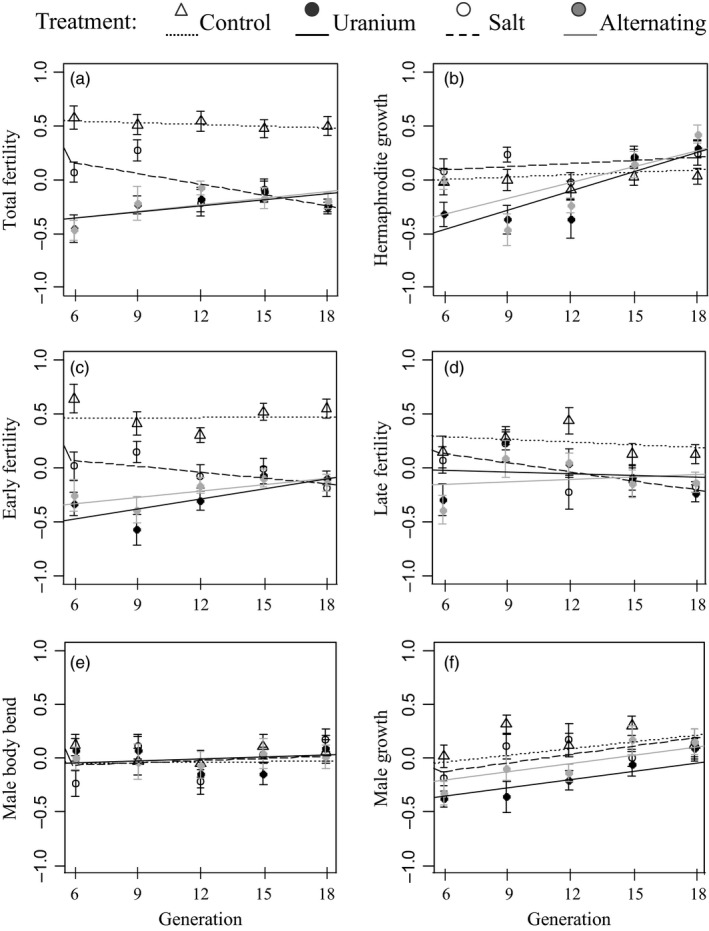
*Caenorhabditis elegans* traits in a nonpolluted common‐garden environment experiment. Responses were measured every three generations beginning at generation 6 of the selection experiment. Traits measured include for hermaphrodites: (a) total fertility, (b) growth, (c) early fertility, (d) late fertility and for males: (e) body bend and (f) growth. Symbols represent means and standard errors for three randomly sampled individuals from each of the six replicates (total 18 individuals per treatment). Traits were rescaled prior to analysis by subtracting each value by the mean of the sample and dividing it by twice the standard deviation. Regression lines correspond to the intercept and the slope posterior modes for each treatment distribution: control* *=* *small dashed line, uranium* *=* *black line, salt* *=* *large dashed line and alternating U/NaCl treatment* *=* *grey line

Between the successive CG experiments, we observed phenotypic changes in hermaphrodite and male traits over time (Figure 2 and Table 2). For hermaphrodite traits, the best‐fitting model included an interaction between treatment and generation and showed that there was covariance between the traits. None of the traits measured in the control populations changed with time (95% HPDIs overlapped 0, Table B1 in Appendix S2). Total fertility for the first CG generation was similar in the NaCl and C populations (Table B1 in Appendix S2), but decreased in the NaCl medium as the number of generations rose: −2.8% per generation relative to C populations (Figure 2a and Table B1 in Appendix S2). In contrast, total fertility was lower in both U and U/NaCl populations in the first CG generation, but it rose steadily as the number of generations increased. The slope for total fertility in U barely overlapped 0 (Table B1 in Appendix S2: 95% HPDI* *=* *[−0.005; 0.052]), but there was a significant positive slope for early fertility (Table B1 in Appendix S2: 95% HPDI* *=* *[0.002; 0.061]): 3.1% per generation relative to C populations (Figure 2c and Table B1 in Appendix S2). In the first CG experiment, growth was lower in the U and U/NaCl populations compared to C populations, but it increased strongly over time: 5.6% and 4.5% per generation, respectively, for the U and U/NaCl populations relative to C populations (Figure 2b and Table B1 in Appendix S2).

The best‐fitting model for male traits included treatment and generation, but not the interaction between them (Table 2). Male growth during the first CG experiment was lower in the U and U/NaCl populations, but growth increased for all populations with time (Figure 2f and Table B1 in Appendix S2). We did not find any treatment or generation effects on body bend frequency (Figure 2e and Table B1 in Appendix S2) or on sex ratio (data not shown for the CG experiments but see Fig. C3 and Table C3 in Appendix S3 for the RT experiment).

### 3.3 Reciprocal‐transplant experiment

**Figure 3:**
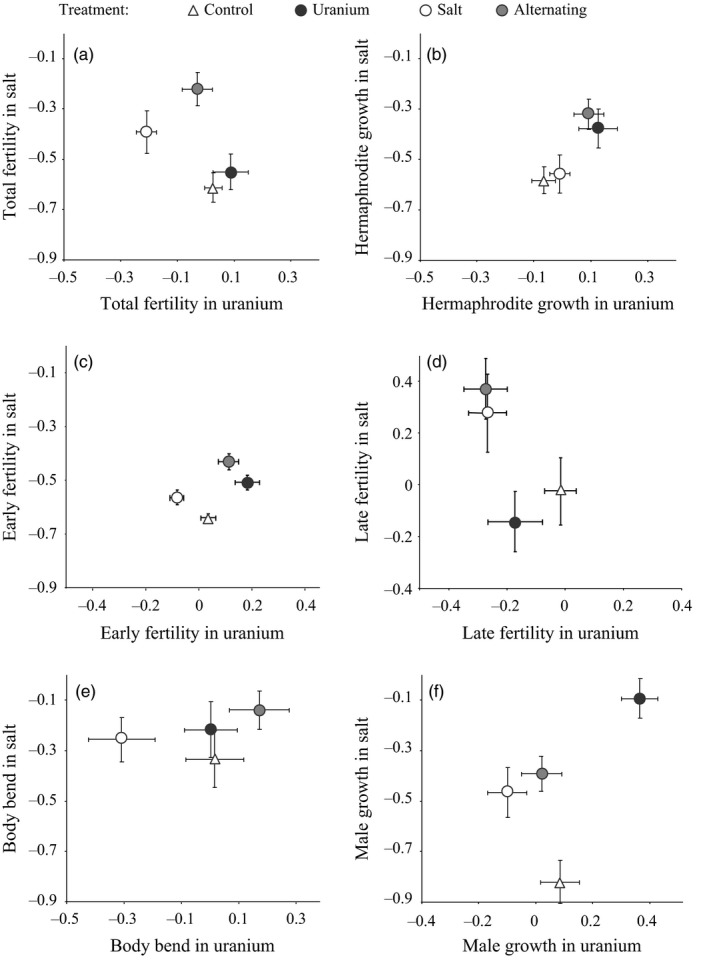
Average traits values in the reciprocal‐transplant experiment. Average values and their standard errors (*n* = 18 individuals) for populations evolved over 18 generations in four different treatments (control, U, NaCl and alternating U/NaCl) and then assessed in environments that have been polluted by either U (*x*‐axis) or NaCl (*y*‐axis). These transplants occurred in the reciprocal‐transplant experiment and at generation 18 of the multigeneration experiment. Traits (rescaled prior to analysis by subtracting each value by the mean of the sample and dividing it by twice the standard deviation) were measured after individuals had spent three generations in the novel environment (i.e., the generation 4). Traits: total fertility (a), hermaphrodite growth (b), early fertility (c), late fertility (d), male body bend (e), male growth (f). Treatment: control* *=* *empty triangle, uranium* *=* *filled black dots, salt* *=* *empty dots, alternating U/NaCl treatment* *=* *filled grey dots

The best‐fitting model for hermaphrodite traits included an interaction between the treatment and the novel environment and showed that there was covariance between traits (Table 3). The environment in which the populations had previously evolved influenced trait expression in the novel environment. The results showed that total fertility for the U and NaCl treatment populations was different (Figure 3a and Table B2 in Appendix S2). In the transplant NaCl environment, NaCl populations had higher total fertility values than the C (total fertility in C minus in NaCl: −0.244 with 95% HPDI* *=* *[−0.371; −0.067]) and U (total fertility in U minus in NaCl: −0.140 with 95% HPDI* *=* *[−0.315; −0.010]) populations. In the transplant U environment, the NaCl populations showed a lower total fertility than C, U and U/NaCl populations (respectively total fertility in C minus in NaCl, U minus NaCl and U/NaCl minus NaCl: 0.209 with 95% HPDI* *=* *[0.082; 0.385], 0.309 [0.139; 0.436] and 0.159 [0.006; 0.318]), but there were no differences between these other three population types. The same pattern was found for late fertility in the transplant NaCl environment, but in the transplant U environment the only significant difference was the lower late fertility for NaCl populations compared to C populations (Figure 3d and Table B2 in Appendix S2). In the transplant U environment, early fertility was lower for NaCl populations (early fertility in C minus in NaCl: 0.113 with 95% HPDI* *=* *[0.031; 0.210]), intermediate for C populations and higher for U populations (early fertility in C minus inU: −0.143 with 95% HPDI* *=* *[−0.234; −0.047]; Figure 3c and Table B2 in Appendix S2). In contrast, in the transplant NaCl environment, early fertility was lower in C populations than in NaCl, U and U/NaCl populations (respectively, early fertility in C minus in U, C minus Na and C minus U/NaCl: −0.115 with 95% HPDI* *=* *[−0.231; −0.040], −0.100 [−0.173; 0.012] barely overlapped 0 and −0.214 [−0.295; −0.113]). Populations that had evolved in the U/NaCl environment showed higher fertility than NaCl populations in the transplant U environment (total fertility in NaCl minus in U/NaCl: −0.159 with 95% HPDI* *=* *[−0. 318; −0. 006]) and than U populations in the transplant NaCl environment (total fertility in U minus in U/NaCl: −0.324 with 95% HPDI* *=* *[−0.478; −0.177]). In both the transplant U and NaCl environments, hermaphrodite growth was higher in U and U/NaCl populations than in C populations and even higher than in NaCl populations in the transplant NaCl environment (Figure 3b and Table B2 in Appendix S2).

The best‐fitting model for male traits included an interaction between the treatment and the novel environment, but there was no covariance between traits (Table 3). In the transplant U environment, NaCl populations had lower body bend frequencies than C, U and U/NaCl populations, but there were no differences between these other three population types (Figure 3e and Table B2 in Appendix S2). There was no difference in body bend frequency or sex ratio between the different types of population in the transplant NaCl environment (Fig. C3 in Appendix S3). In both the transplant NaCl and U environments, U populations produced larger males than C, NaCl and U/NaCl populations (Figure 3f and Table B2 in Appendix S2). Overall, the control populations produced the smallest males when exposed to the NaCl environment.

## 4 DISCUSSION

We found that *C. elegans* populations could rapidly adapt to both the uranium and salt environments (fitness increased between generation 4 and the end of the selection experiment: Figure 1 and Table 1). Adaptation to uranium or to salt was associated with some fitness costs, as shown by their lower fertility values compared to the control populations (i.e., into the uranium environment for NaCl populations: Figure 3a, c and Table B2 in Appendix S2; into the original environment for U and NaCl populations: Figure 2a, c and Table B1 in Appendix S2). However, adaptation costs did not seem to be systematic, and adaptation to uranium appeared to be beneficial in the NaCl environment (Figure 3b, c, f and Table B2 in Appendix S2). Furthermore, adaptation to the alternating environment appeared to confer similar or even higher trait values than those for populations that had evolved in response to only one type of stressor (Figure 3a and Table B2 in Appendix S2). These results indicate that adaptation to different environments can lead to both fitness costs and benefits. Finally, with only one CG experiment, we would have missed the genetic differentiation in fertility in the salt compared to the control environment or in growth in the uranium compared the control environment (Figure 2a, b and Table B1 in Appendix S2). The absence of evidence for genetic differentiation between populations at one particular time, as is generally done with natural populations, does not guarantee that the two populations are not actually diverging: only repeated CG experiments can show the dynamics of genetic differentiation over time.

### 4.1 Life history and fitness responses to differential selection pressures

The populations subjected to each of the three polluted environments showed a strong decrease in fitness during the first generations and a fast increase afterwards (Figure 1 and see Dutilleul et al., 2014 for more results on the selection experiments). Differences in the slopes of the trait values with generation in the successive CG experiments revealed evolutionary (i.e., genetic) responses to the three polluted environments (Table B1 in Appendix S2). Our results also indicate that populations responded to the different stressors with different life history adaptations.

In response to salt exposure, an extreme hypertonic stress, *C. elegans* regulates the rapid loss of water and solute content in its cells by synthesizing glycerol through transcriptional upregulation of an enzyme (gpdh‐1) in the intestine and hypodermis, two tissues that are both in direct contact with the external medium (Lamitina, Huang, & Strange, 2006; Lamitina et al., 2004). In our experiment, *C. elegans* populations responded to salt by reducing early and total fertility (Figure 2), thereby producing individuals with longer life cycles and lower fertility. Moreover, NaCl populations showed reduced survival compared to both uranium and control treatments (see Fig. C and Table C2 in Appendix S3). Consequently, survival may be more essential than the rapid production of a large number of embryos for the NaCl populations (e.g., an increase in maintenance costs because energy is diverted from reproduction towards water regulation).

Uranium populations showed survival similar to control populations, throughout the selection experiment (see Dutilleul et al., 2014), and in the RT experiment (Fig. C and Table C2 in Appendix S3); however, fertility and growth of U or U/NaCl populations showed a strong decline in the first CG experiment. However, this decline was followed by an evolutionary increase in fertility (in particular early fertility) and growth in later CG experiments (Figure 2). When transplanted to either the uranium or salt environments, the U populations grew faster than the control and NaCl populations (Figure 3). To summarize, uranium selects for fast growth, high early fertility and thus a fast generation time. Growing faster and becoming larger may allow individuals to detoxify their bodies, prevent internalization of the pollutant and reduce internal pollutant concentrations (Guedes et al., 2006; Sibly & Calow, 1989). For example, uranium severely affects the intestinal epithelium in the earthworm, *Eisenia fetida* (Giovanetti et al., 2010) and seems to decrease energy assimilation in *C. elegans* (Goussen et al., 2015). The presence of uranium in the environment increases the expression of metallothionein‐1 (mtl‐1), which interferes with U accumulation in cells, probably by sequestering and removing uranium from the cells (Jiang et al., 2009). Uranium seems to be associated with a rapid life cycle that helps reduce the period of contact with the pollutant. However, for both pollutants, the reallocation of energy is performed at the expense of other traits or fitness in other environments (Hoffmann & Parsons, 1991; Reznick et al., 2000).

Consequently, salt‐adapted populations seemed to have evolved towards slower life history strategies, whereas uranium and alternating U/NaCl populations seemed to have evolved towards faster life histories. Pollutants may thus have strong consequences on the evolution of populations along the fast–slow life history continuum (Promislow & Harvey, 1990; Stearns, 1983) with potentially strong implications for their dynamics. Such opposite selection pressures between NaCl and U may lead to maladaptation to the other environment. Maladaptation is suggested by the lower total fertility of NaCl populations in uranium and of U populations in NaCl (Figure 3a). Alternating populations, however, do not seem to show such maladaptive outcomes, which indicates that evolving in a more heterogeneous environment may help populations dealing with future environmental changes. It should also be noted that all the life history traits that we measured were phenotypically correlated with each other. Phenotypic integration (i.e., functionally related traits are correlated) between the studied traits (Pigliucci & Preston, 2004) may lead to coevolution of the traits associated to a particular life history strategy.

### 4.2 Costs and benefits of adaptation

After 22 generations of steady exposure, NaCl populations showed lower fertility, male growth and body bend in the uranium than U or C populations (Figure 3). These results indicate that evolution to salt bears a fitness cost in terms of tolerance to uranium. Furthermore, when they were returned to the control environment, U, NaCl and U/NaCl populations showed lower fertility than the control populations (Figure 2), indicating adaptation costs incurred by the adaptive evolution in a polluted environment. Previous studies have also shown adaptation costs associated with evolution in response to pollutants (Jansen, Stoks, et al., 2011; Mireji et al., 2010; Shirley & Sibly, 1999; Ward & Robinson, 2005; Xie & Klerks, 2003 but see Coustau et al., 2000; Reznick et al., 2000; McCart et al., 2005; Lopes et al., 2008). Such costs limit the ability of polluted populations to deal with their new environmental conditions once the environment is depolluted. Negative cross‐environment genetic correlations caused by antagonistic pleiotropic effects are assumed to be at the origin of adaptation costs (Falconer & Mackay, 1996; Fry, 1993). In a previous study of “isogenic” lines of *C. elegans*, we did not find any negative cross‐environment genetic correlations for the traits under study (Dutilleul, Goussen, Bonzom, Galas, & Réale, 2015). In a stressful environment, however, the expression of genes governing quantitative traits can be masked by the action of genes involved in detoxification (Hoffmann & Parsons, 1991). Indeed, *C. elegans’* tolerance to several heavy metals, including uranium, is related to one or a few major genes (Aschner & Martinez‐Finley, 2011). These genes with major effects may mask the expression of genes for quantitative traits, such as life history traits. Nonetheless, selection may still act on these traits. Adaptations to pollutants include a reduction in pollutant assimilation (Xie & Klerks, 2003), increased pollutant excretion (Lagauzère, Terrail, & Bonzom, 2009; Posthuma & Van Straalen, 1993) and pollution sequestration (e.g., metallothionein synthesis; Gillis, Diener, Reynoldson, & Dixon, 2002; Jiang et al., 2009; Shirley & Sibly, 1999). These mechanisms are assumed to be nonplastic and cannot be shut down if the environment becomes unpolluted again (Morgan et al., 2007). In the absence of that pollutant, these mechanisms are energetically costly to maintain and, thus, become disadvantageous.

Changes in life history traits observed in response to pollutants probably reflect changes in these detoxifying mechanisms at the molecular, biochemical and physiological levels. Selection may favour genotypes that allocate more resources to detoxification at the expense of other fitness‐related functions, with some important life history consequences. In such case, life history evolution may represent a by‐product of the evolutionary changes of populations towards an increasing allocation to detoxifying functions. Once the pollutant is removed, however, energetic costs related to these detoxification activities still affect life history and fitness (Burdon & Thrall, 2003; Kraaijeveld & Godfrey, 1997). Alternatively, selection caused by the pollutant may act directly on some life history or behavioural traits, because the genotypes favoured by selection experience reduced impact from the pollutants. For example, compared to slow‐growing genotypes, fast‐growing precocious genotypes can minimize exposure to a pollutant before they start reproducing, therefore minimizing the impact of the pollutant on their fitness (Sibly & Calow, 1989). Our results suggest that this is what has happened in the uranium environment.

Following strong pollution, selection pressures may reduce genetic diversity over the short term at a faster rate than mutations can generate new diversity (Athrey et al., 2007; Nowak et al., 2009; Ward & Robinson, 2005). The low variance among replicate populations in the selection experiment (< 4% of the total trait variance, see Dutilleul et al., 2014 for more details) indicates that genetic drift generated negligible random divergence among replicates for the studied traits. We can therefore expect that selection will act mainly on a population's standing genetic variation (Denver et al., 2009; Mackay, Fry, Lyman, & Nuzhdin, 1994) and that reductions in genetic diversity may be partly responsible for the adaptation costs observed in our study. However, phenotypic variance for the studied traits does not seem to be lower in pollution‐adapted populations than in the control populations (see Figure 3), which may indicate that rather than a decrease in genetic variance, antagonistic pleiotropy is largely responsible for the adaption costs observed in our experiments.

Adaptation costs also seem to depend on the type of pollutant. For example, in the RT experiment, NaCl populations showed signs of specialization and of adaptation costs, but that was not the case for U populations (Figure 3a, b, c, f). Several studies have shown asymmetric adaptation costs in viruses (Kassen, 2002; Kraaijeveld & Godfrey, 1997 and references therein; Jasmin & Kassen, 2007). This asymmetry must correspond to the different pleiotropic effects associated with changes in response to different novel environmental conditions (Jasmin & Kassen, 2007; Rose et al., 2005; Travisano & Lenski, 1996).

Finally, the adaptation costs identified in this study were not systematic: some traits did not show any adaption costs (e.g., U populations in Figure 3a) or even showed higher values in another polluted environment (e.g., U populations in Figure 3b, c, f). These results indicate potential cross‐resistance to the different pollutants, and that adaptation to one particular environment leads to selective benefits in another environment. Cross‐resistance to heavy metals (e.g., cadmium and lead or cadmium and copper), or insecticides, has been found previously in plants, invertebrates and fish (McKenzie, 1996; Ward & Robinson, 2005; Watmough & Dickinson, 1995; Xie & Klerks, 2003). To explain the beneficial effects, these researchers assumed that detoxification mechanisms were common to the different pollutants. This could be possible when there is a single major gene or a few genes with effects specific to a class of pollutants or even to more general actions of pollutants (McKenzie, 1996; Xie & Klerks, 2003). Knowledge on biochemical, physiological and molecular mechanisms that are associated with the adaptation to each pollutant would help us predict when populations may be able to adapt to a series of different pollutants and in which combination of pollutants adaptive costs or benefits are expected.

### 4.3 Changing environments and the evolution of generalism

It is recognized that fluctuating or changing environments promote generalist genotypes and that constant environments promote specialist genotypes (Cooper & Lenski, 2000; Reboud & Bell, 1997; Turner & Elena, 2000). In both transplant U and NaCl environments, U/NaCl populations showed similar or better early and total fertility than both U and NaCl populations (Figure 3). Our results thus confirm the hypothesis that alternating or changing environmental conditions produce generalist genotypes that could have an advantage when exposed to a single pollutant. Similar results have been found in other systems. Populations of viruses exposed to an alternating regime of two different hosts were as well adapted to each host as populations adapted to a single host (Turner & Elena, 2000). Moreover, fluctuating or changing environments have been shown to promote more within‐population diversity than stable ones (Buckling, Kassen, Bell, & Rainey, 2000; Collins, 2011; Cooper & Lenski, 2010). Reed et al. (2003) have shown enhanced fitness in a novel polluted environment for *Drosophila melanogaster* populations adapted to two alternating stressors compared to populations adapted to only one of them. These results could change the classical vision that adaptation to several stressors decreases the adaptive potential of populations (Hoffmann & Parsons, 1991; Koskella et al., 2012; Tilman & Lehman, 2001). Experimental evolution studies should thus mimic more precisely the temporally and spatially heterogeneous environments found in natural conditions to provide a better understanding of the adaptive potential of populations to pollutants.

### 4.4 Implications for natural populations in polluted environments

Understanding the mechanisms and evolutionary consequences for populations subjected to sources of pollution has recently become a major research area because we need to improve ecological risk assessment (ERA) processes (Coutellec & Barata, 2011; Klerks, Xie, & Levinton, 2011). Our results confirm the existence of trade‐offs between adaptation to a particular stressor and the capacity of a population to cope with other future stressors (Coustau et al., 2000; Roff & Fairbairn, 2007). Furthermore, we have shown that adaptive costs can appear very quickly in the presence of a pollutant (Jansen, Stoks, et al., 2011; Salice et al., 2010; Xie & Klerks, 2003). In many cases, pollution can increase rapidly and decrease (e.g., reduction of the discharges, degradation or dilution of the pollutants in the ecosystem) and can vary strongly in space (Medina, Correa, & Barata, 2007; Morgan et al., 2007). Populations may quickly adapt to high pollution peaks but then will not be able to cope with a return to original conditions once the pollutions cease, or will be less able to adapt to a new stressor. Thus, the interaction of anthropogenic stressors with other selection pressures may have rapid and severe consequences on natural populations.

A very large number of pollutants or stressors can lead to completely different and opposite life history strategies. Consequently, it is a challenge to anticipate the evolutionary consequences of chemicals produced by industry or anthropogenic stressors as they may drive the populations in totally different directions and increase their risk of extinction. One important application of an experimental evolutionary approach to the ERA may be to experimentally expose some model species to a variety of pollutants that have known effects on the organisms, so that we can develop a general classification of evolutionary responses by organisms to different classes of pollutants. The classification could then be used as a tool to better anticipate the risk posed by pollutants. However, because species are rarely exposed to a single pollutant or stressor in time and space, the mixture risk assessment remains a significant challenge. In addition, the implementation of the evolutionary endpoints in environmental risk assessment of pollutants and regulatory decision‐making remains an important issue.

## DATA ARCHIVING STATEMENT

The data sets supporting the results of this article are available from the Dryad Digital Repository: https://doi.org/10.5061/dryad.17tf0.
