# Supplementary material for: Adaptation costs to constant and alternating polluted environments
Source: Evol Appl. 2017 Nov 10;10(8):839–51. doi: 10.1111/eva.12510 (PMC5680423; doi:10.1111/eva.12510)

Appendix S1: Schematic overview of the experiments

Figure A. Representation of the generations (G : generation) where a part of each population from the selection experiment were used to conduct the common garden and reciprocal transplant experiments (A1) and representation of the experiments of common garden, CG (A2), reciprocal transplant, RT (A3). In each experiment, populations were kept in their novel environment for three generations prior to measuring the traits (500 individuals per replicate and 6 replicates per environment). The CG experiments allow to test (1) whether adaptation to a particular pollutant incurs adaptation costs when the population is in a non-polluted environment, the RT experiment allows to test (2) whether adaptation to a particular pollutant incurs adaptation costs when the population is subjected to another stressor, and the RT and the CG experiments both allow to test (3) whether adaptation costs differ between heterogeneous and homogenous environments.

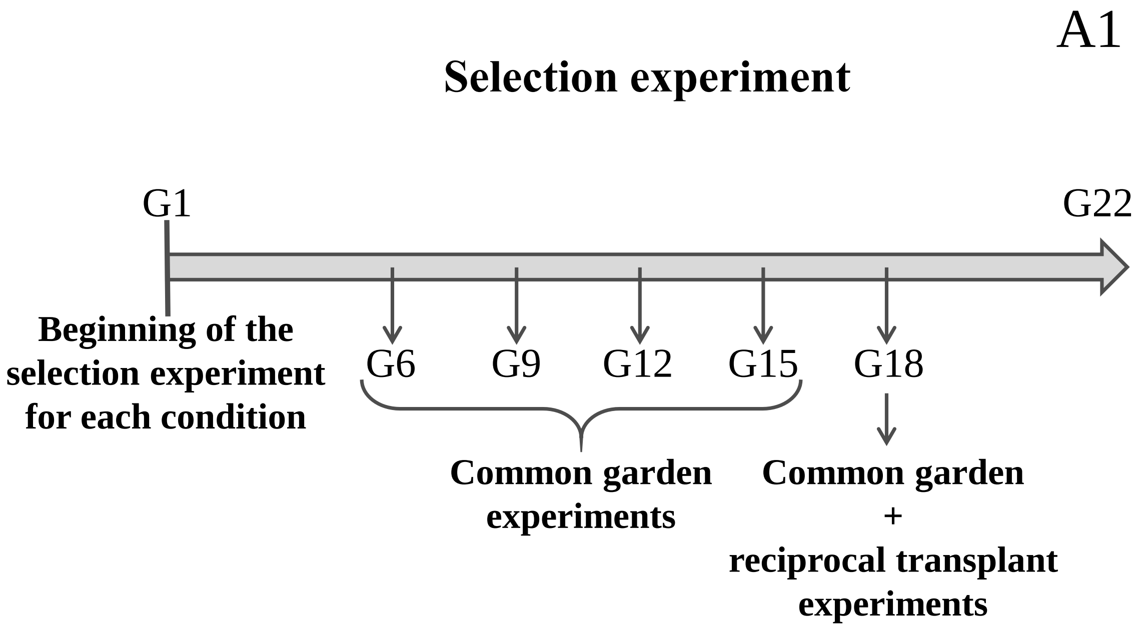

A2

Common garden experiments

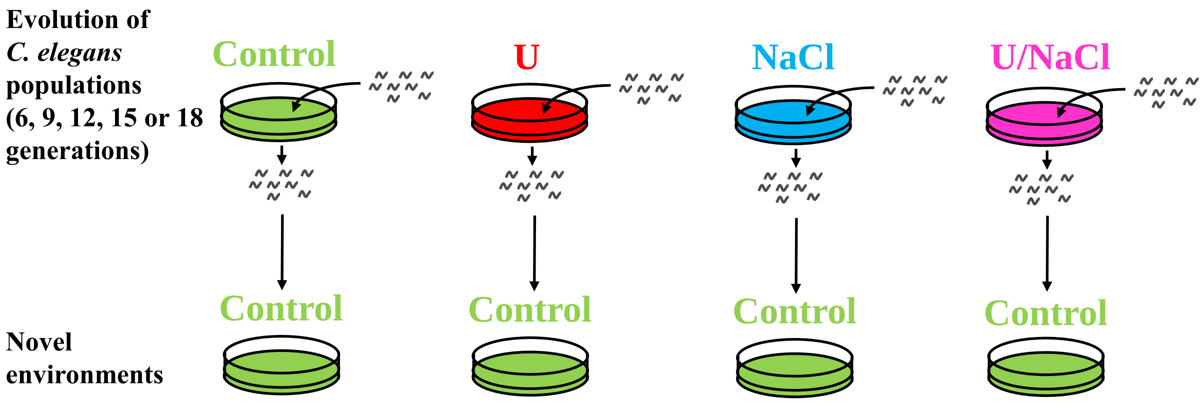

A3

Reciprocal transplant experiment

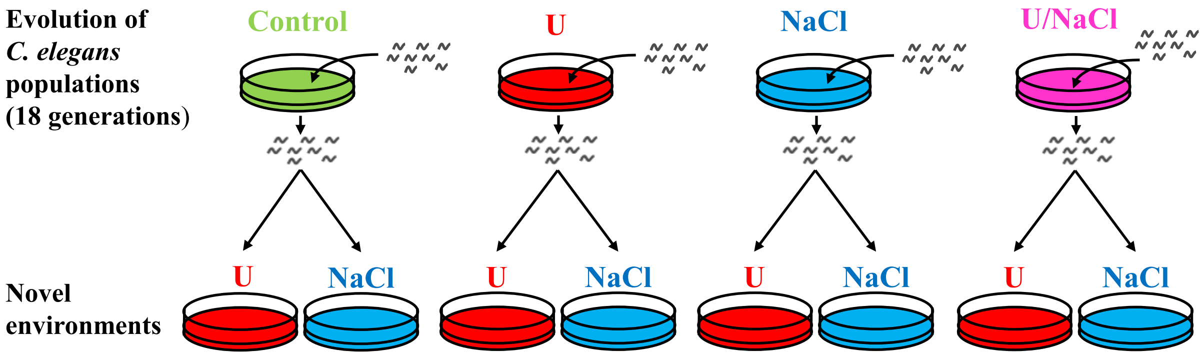

Supplement: Supplementary file 1 [file EVA-10-839-s001.pdf]
