# Supplementary material for: Adaptation costs to constant and alternating polluted environments
Source: Evol Appl. 2017 Nov 10;10(8):839–51. doi: 10.1111/eva.12510 (PMC5680423; doi:10.1111/eva.12510)
Supplement: Supplementary file 2 [file EVA-10-839-s002.pdf]

Appendix S2: Statistics tables of the generalized linear mixed effects models

Table B1. Difference in *C. elegans* hermaphrodite (A) and male (B) trait values for five successive common-garden experiments (generations 6, 9, 12, 15 and 18) along with the multi-generation experiment. Intercept corresponds to the trait values obtained from the first common-garden (generation 6) experiment and slope is the linear regression slope as the number of generations increases. Values correspond to the intercept and slope posterior modes of the distribution in control or relative to the control in the treatments (U, NaCl and U/NaCl), except for the male trait slope (one estimation for all treatments). Traits were rescaled prior to analysis by subtracting each value by the mean of the sample and dividing it by twice the standard deviation. Values between brackets: 95% highest posterior density interval (HPDI). In bold: values where the 95% HPDI did not overlap 0.

A

Parameter analysed	Control		Uranium		Salt		Alternating U/NaCl	
Intercept								
Total fertility	**0.596**	**[0.255 ; 0.911]**	**-1.121**	**[-1.615 ; -0.690]**	-0.145	[-0.655 ; 0.260]	**-1.130**	**[-1.558 ; -0.690]**
Early fertility	**0.451**	**[0.140 ; 0.822]**	**-1.206**	**[-1.738 ; -0.775]**	-0.214	[-0.674 ; 0.267]	**-0.961**	**[-1.445 ; -0.507]**
Late fertility	0.365	[-0.063 ; 0.763]	-0.331	[-0.917 ; 0.152]	0.014	[-0.537 ; 0.547]	**-0.584**	**[-1.118 ; -0.060]**
Growth	-0.071	[-0.438 ; 0.289]	**-0.947**	**[-1.450 ; -0.464]**	0.070	[-0.472 ; 0.566]	**-0.697**	**[-1.196 ; -0.156]**
Slope								
Total fertility	-0.005	[-0.026 ; 0.016]	0.024	[-0.005 ; 0.052]	**-0.028**	**[-0.057 ; -0.002]**	**0.026**	**[0.001 ; 0.053]**
Early fertility	0.001	[-0.021 ; 0.021]	**0.031**	**[0.002 ; 0.061]**	-0.019	[-0.049 ; 0.009]	0.019	[-0.011 ; 0.049]
Late fertility	-0.008	[-0.032 ; 0.019]	0.003	[-0.030 ; 0.039]	-0.019	[-0.054 ; 0.015]	0.016	[-0.019 ; 0.048]
Growth	0.004	[-0.017 ; 0.029]	**0.056**	**[0.026 ; 0.088]**	0.006	[-0.026 ; 0.039]	**0.045**	**[0.013 ; 0.080]**

B

Parameter analysed	Control		Uranium		Salt		Alternating U/NaCl	
Intercept								
Growth	-0.226	[-0.431 ; -0.015]	**-0.359**	**[-0.548 ; -0.209]**	-0.127	[-0.318 ; 0.027]	**-0.227**	**[-0.393 ; -0.071]**
Body Bend	-0.044	[-0.269 ; 0.175]	-0.051	[-0.223 ; 0.130]	-0.072	[-0.243 ; 0.102]	-0.047	[-0.215 ; 0.135]
Slope (for all treatments)								
Growth	**0.027**	**[0.016 ; 0.038]**	-		-		-	
Body Bend	0.006	[-0.007 ; 0.018]	-		-		-	

Table B2. Differences in *C. elegans* hermaphrodite and male traits values between the populations that had previously been exposed to a uranium, salt or alternating uranium and salt environment, and had been transplanted to a novel environment (i.e. uranium or salt). Values correspond to the posterior mode of the distribution of trait differences between pairs of populations from different treatments. Traits were rescaled prior to analysis by subtracting each value by the mean of the sample and dividing it by twice the standard deviation. Values between brackets: 95% highest posterior density interval (HPDI). In bold: values where the 95% HPDI did not overlap 0.

Comparison	Hermaphrodite traits								Male traits			
	Total fertility		Early fertility		Late fertility		Growth		Body bend		Growth	
In uranium												
Control-Uranium	-0.086	[-0.193 ; 0.107]	**-0.143**	**[-0.234 ; -0.047]**	0.212	[-0.100 ; 0.419]	**-0.201**	**[-0.347 ; -0.050]**	0.005	[-0.246 ; 0.261]	**-0.263**	**[-0.465 ; -0.077]**
Control-Salt	**0.209**	**[0.082 ; 0.385]**	**0.113**	**[0.031 ; 0.210]**	**0.296**	**[0.019 ; 0.549]**	-0.077	[-0.196 ; 0.108]	**0.261**	**[0.073 ; 0.586]**	0.176	[-0.025 ; 0.396]
Control-Alternating	0.039	[-0.082 ; 0.224]	-0.075	[-0.167 ; 0.019]	0.193	[-0.005 ; 0.531]	**-0.177**	**[-0.313 ; -0.016]**	-0.123	[-0.428 ; 0.104]	0.074	[-0.134 ; 0.271]
Uranium-Salt	**0.309**	**[0.139 ; 0.436]**	**0.255**	**[0.176 ; 0.354]**	0.073	[-0.148 ; 0.369]	0.137	[-0.018 ; 0.281]	**0.296**	**[0.042 ; 0.558]**	**0.449**	**[0.255 ; 0.657]**
Uranium-Alternating	0.112	[-0.037 ; 0.267]	0.071	[-0.022 ; 0.159]	0.082	[-0.138 ; 0.386]	0.018	[-0.112 ; 0.188]	-0.159	[-0.402 ; 0.136]	**0.320**	**[0.159 ; 0.546]**
Salt-Alternating	**-0.159**	**[-0.318 ; -0.006]**	**-0.195**	**[-0.279 ; -0.102]**	0.113	[-0.240 ; 0.308]	-0.127	[-0.269 ; 0.043]	**-0.414**	**[-0.753 ; -0.209]**	-0.158	[-0.323 ; 0.088]
In salt												
Control-Uranium	-0.064	[-0.205 ; 0.088]	**-0.115**	**[-0.231 ; -0.040]**	0.139	[-0.126 ; 0.385]	**-0.222**	**[-0.366 ; -0.055]**	-0.041	[-0.396 ; 0.140]	**-0.708**	**[-0.932 ; -0.514]**
Control-Salt	**-0.244**	**[-0.371 ; -0.067]**	-0.100	[-0.173 ; 0.012]	**-0.321**	**[-0.588 ; -0.062]**	-0.038	[-0.178 ; 0.123]	-0.015	[-0.367 ; 0.189]	**-0.352**	**[-0.554 ; -0.172]**
Control-Alternating	**-0.403**	**[-0.533 ; -0.249]**	**-0.214**	**[-0.295 ; -0.113]**	**-0.393**	**[-0.641 ; -0.134]**	**-0.272**	**[-0.415 ; -0.103]**	-0.181	[-0.459 ; 0.078]	**-0.427**	**[-0.630 ; -0.235]**
Uranium-Salt	**-0.140**	**[-0.315 ; -0.010]**	0.046	[-0.034 ; 0.161]	**-0.443**	**[-0.667 ; -0.120]**	**0.189**	**[0.030 ; 0.338]**	0.050	[-0.229 ; 0.299]	**0.420**	**[0.186 ; 0.587]**
Uranium-Alternating	**-0.324**	**[-0.478 ; -0.177]**	-0.096	[-0.165 ; 0.016]	**-0.515**	**[-0.744 ; -0.220]**	-0.045	[-0.204 ; 0.107]	-0.098	[-0.356 ; 0.173]	**0.287**	**[0.091 ; 0.507]**
Salt-Alternating	**-0.139**	**[-0.324 ; -0.012]**	**-0.141**	**[-0.222 ; -0.034]**	-0.057	[-0.350 ; 0.194]	**-0.215**	**[-0.383 ; -0.072]**	-0.201	[-0.394 ; 0.158]	-0.065	[-0.260 ; 0.131]
