# Supplementary material for: Adaptation costs to constant and alternating polluted environments
Source: Evol Appl. 2017 Nov 10;10(8):839–51. doi: 10.1111/eva.12510 (PMC5680423; doi:10.1111/eva.12510)
Supplement: Supplementary file 3 [file EVA-10-839-s003.pdf]

Appendix S3: Representation of the percentage of survival until 48h

Figure C. Survival percentage (C1, C2) during the common-garden experiments (C1) and reciprocal-transplant experiment (C2), and male percentage (C3) during the reciprocal-transplant experiment. Symbols represent the mean and its associated standard error for six replicates with approximatively 100 individuals randomly sampled in each treatment. Control = empty triangle; uranium = filled black dots; salt = empty dots; alternating U/NaCl treatment = filled grey dots.

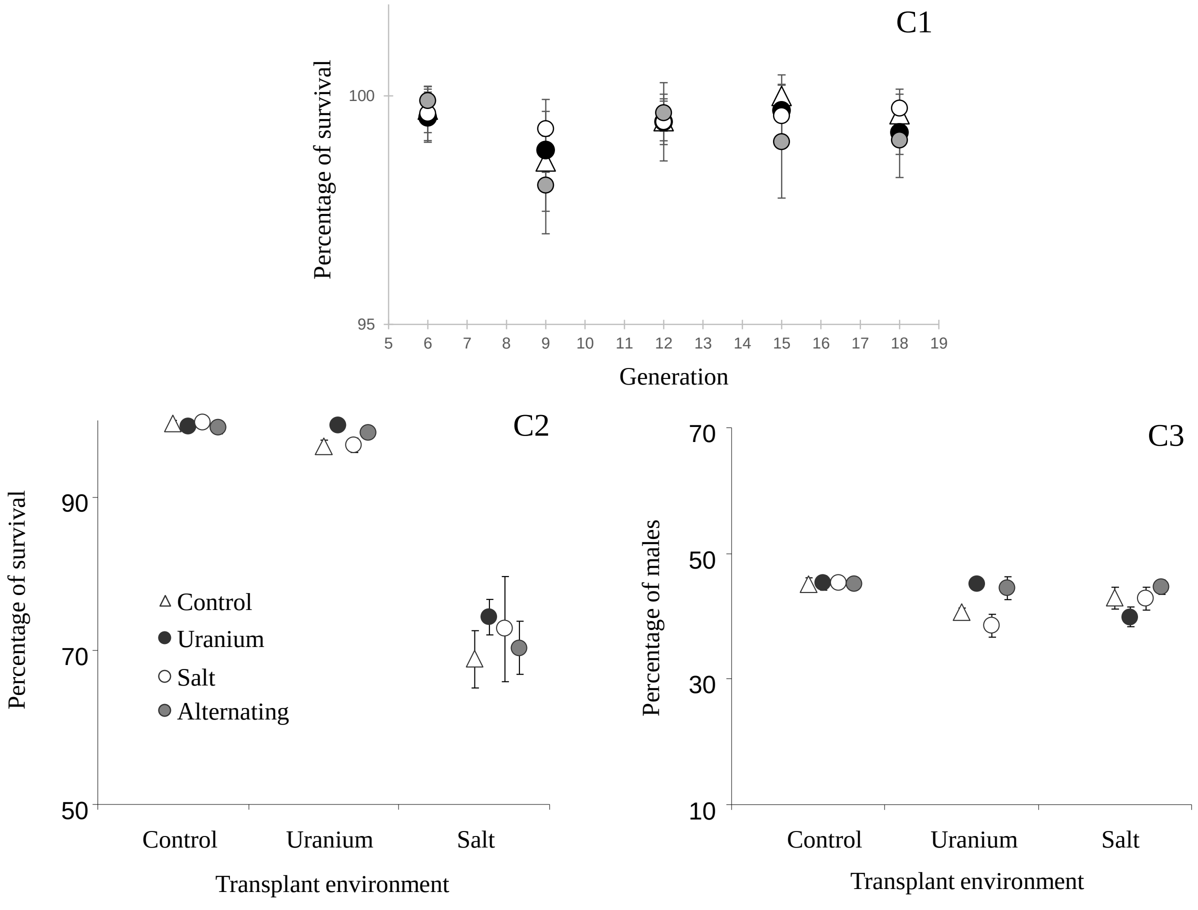

Table C1. Analysis of deviance for a generalized linear model (binomial distribution) for the response of survival at 48h measured in common-garden experiments in a control environment at generation 6, 9, 12, 15 and 18.

Source	*df*	*Residual deviance*	*P (chi-square)*
-	119	166.06	
generation	1	165.91	0.698
treatment	3	162.84	0.382
generation × treatment	3	161.43	0.703

df: degree of freedom; *P*: probability of significance obtain by chi-squared test comparing the reduction in deviance for the row to the residuals.

Table C2. Analysis of deviance for a generalized linear model (binomial distribution) for the response of survival at 48h measured in the reciprocal-transplant experiment at generation 18.

Source	*df*	*Residual deviance*	*P (chi-square)*
-	71	64.881	
transplant environment	2	46.369	***
treatment	3	45.310	0.7868
transplant environment × treatment	6	36.639	0.1930

df: degree of freedom; *P*: probability of significance obtain by chi-squared test comparing the reduction in deviance for the row to the residuals.

Table C3. Analysis of deviance for a generalized linear model (binomial distribution) for the response of male percentage measured in the reciprocal-transplant experiment at generation 18.

Source	*df*	*Residual deviance*	*P (chi-square)*
-	71	10.539	
transplant environment	2	8.314	0.329
treatment	3	5.407	0.406
transplant environment × treatment	6	5.407	1.000

df: degree of freedom; *P*: probability of significance obtain by chi-squared test comparing the reduction in deviance for the row to the residuals.
